# In vivo calibration of esophageal pressure in the mechanically ventilated patient makes measurements reliable

**DOI:** 10.1186/s13054-016-1278-5

**Published:** 2016-04-11

**Authors:** Francesco Mojoli, Giorgio Antonio Iotti, Francesca Torriglia, Marco Pozzi, Carlo Alberto Volta, Stefania Bianzina, Antonio Braschi, Laurent Brochard

**Affiliations:** Anesthesia and Intensive Care, Emergency Department, Fondazione IRCCS Policlinico S. Matteo, Pavia, Italy; Anesthesia, Intensive Care and Pain Therapy, Department of Clinical, Surgical, Diagnostic and Pediatric Sciences, University of Pavia, Pavia, Italy; Anesthesia and Intensive Care, Department of Morphology, Surgery and Experimental Medicine, University of Ferrara, Arcispedale Sant’Anna, Ferrara, Italy; Keenan Research Centre, Li Ka Shing Knowledge Institute, St. Michael’s Hospital, Toronto, ON Canada; Interdepartmental Division of Critical Care Medicine, University of Toronto, Toronto, ON Canada

**Keywords:** Esophageal pressure, Pleural pressure, Transpulmonary pressure, Mechanical ventilation, Protective ventilation, Ventilator-induced lung injury, Calibration, Esophageal balloon catheter, Esophageal elastance, Esophageal artifact

## Abstract

**Background:**

Esophageal pressure (Pes) can provide information to guide mechanical ventilation in acute respiratory failure. However, both relative changes and absolute values of Pes can be affected by inappropriate filling of the esophageal balloon and by the elastance of the esophagus wall. We evaluated the feasibility and effectiveness of a calibration procedure consisting in optimization of balloon filling and subtraction of the pressure generated by the esophagus wall (Pew).

**Methods:**

An esophageal balloon was progressively filled in 36 patients under controlled mechanical ventilation. V_BEST_ was the filling volume associated with the largest tidal increase of Pes. Esophageal wall elastance was quantified and Pew was computed at each filling volume. Different filling strategies were compared by performing a validation occlusion test.

**Results:**

Fifty series of measurements were performed. V_BEST_ was 3.5 ± 1.9 ml (range 0.5–6.0). Esophagus elastance was 1.1 ± 0.5 cmH_2_O/ml (0.3–3.1). Both Pew and the result of the occlusion test differed among filling strategies. At filling volumes of 0.5, V_BEST_ and 4.0 ml respectively, Pew was 0.0 ± 0.1, 2.0 ± 1.9, and 3.0 ± 1.7 cmH_2_O (*p* < 0.0001), whereas the occlusion test was satisfactory in 22 %, 98 %, and 88 % of cases (*p* < 0.0001).

**Conclusions:**

Under mechanical ventilation, an increase of balloon filling above the conventionally recommended low volumes warrants complete transmission of Pes swings, but is associated with significant elevation of baseline. A simple calibration procedure allows finding the filling volume associated with the best transmission of tidal Pes change and subtracting the associated baseline artifact, thus making measurement of absolute values of Pes reliable.

**Electronic supplementary material:**

The online version of this article (doi:10.1186/s13054-016-1278-5) contains supplementary material, which is available to authorized users.

## Background

Esophageal manometry has been proposed as a clinical surrogate for pleural pressure direct measurement, to guide mechanical ventilation in acute respiratory failure [1, 2]. Esophageal pressure (Pes) is currently measured by an esophageal catheter with an air-filled balloon placed in the mid-lower portion of the esophagus. The prerequisite for using this technique is that the pressure surrounding the esophagus, i.e., the intrathoracic pressure, is correctly transmitted to the balloon. Several factors may affect the results [3, 4]. Large-size balloons, only partially inflated, are used to avoid any additional pressure due to balloon wall stretching [5, 6]. Nonetheless, due to the elastance of the esophagus wall, the filling volume was found to affect measured Pes even when the balloon is only partially inflated [5, 7]. Milic-Emili and coworkers [6] proposed two strategies to eliminate this artifact: using very low filling volumes (≤0.5–1.0 ml) or correcting Pes values by extrapolation to zero balloon volume. The first option, simpler, was thereafter adopted by researchers and clinicians, and discussed no further. Only recently, an in vitro study clearly demonstrated that, under positive-pressure conditions like during mechanical ventilation, very low filling volumes may be insufficient to accurately transmit both absolute values and tidal swings of Pes [8]. On the other hand, higher than traditional filling volumes result in disproportionately high Pes values, as recently reported by Chiumello and coworkers [9]. These findings support the concept that only respiratory swings of Pes are reliable, and not absolute values, thus firing up the controversy on alternative methods to compute transpulmonary pressure [10].

With the present study, we propose a new Pes calibration procedure designed to solve both the issue of low Pes transmission due to insufficient balloon filling, and the opposite issue of Pes overestimation due to significant pressure generated by the esophageal wall as a reaction to balloon filling. This procedure was tested on patients with acute respiratory failure (ARF), to evaluate its feasibility and possible advantages over traditional, not calibrated, approaches.

## Methods

We enrolled sedated and paralyzed patients with ARF under pressure-controlled mechanical ventilation, in whom an esophageal balloon catheter (Nutrivent, Sidam, Mirandola, Italy) had been inserted in the mid-lower third of the thoracic esophagus for clinical purposes. The esophageal balloon has a length of 10 cm, a nominal volume of 10 ml and a factory-recommended inflating volume of 4 ml of air. Mechanical characteristics of this catheter and other commercially available ones were previously studied in vitro [8]. Briefly, the devices were exposed to different surrounding pressures and progressively filled with air to obtain esophageal balloon pressure-volume curves. The appropriate range of filling volumes was defined by a null balloon transmural pressure and corresponded, for all the devices, to the intermediate linear section of the curve. The appropriate range was found to be catheter-specific and related to the surrounding pressure, being 0.5–8 ml for the NutriVent catheter when the surrounding pressure was in the 0–30 cmH_2_O range. Appropriate catheter position was confirmed by visualization of cardiac artifacts on Pes and radiopaque markers on chest X-ray.

**Fig. 1:**
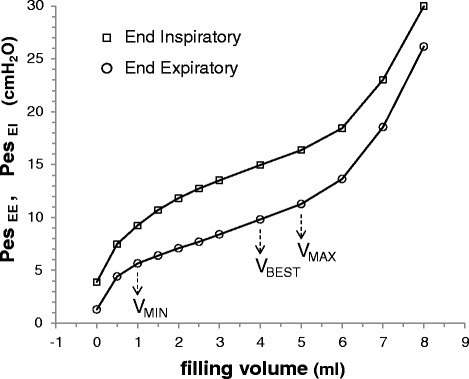
Static esophageal balloon pressure-volume curves. Relationship between balloon filling volume and static values of Pes, both at end-expiration (Pes_EE_, *circles*) and at end-inspiration (Pes_EI_, *squares*). On the end-expiratory pressure-volume (PV) curve, the intermediate linear section was graphically detected and analyzed for its lower and upper limits (V_MIN_ and V_MAX_, respectively). The range between V_MIN_ and V_MAX_ was considered to correspond to appropriate balloon filling, with volumes below V_MIN_ denoting underfilling and volumes above V_MAX_ denoting overdistention. The elastance of the esophagus (cmH_2_O/ml) was considered equivalent to the slope of this section of the end-expiratory PV curve. Within the appropriate filling range, we identified V_BEST_, i.e., the filling volume associated with the maximum difference between Pes_EI_ and Pes_EE_. *Pes* esophageal pressure

In each patient, we recorded the static Pes at end-expiration (Pes_EE_) and end-inspiration (Pes_EI_) while the esophageal balloon was inflated with increasing volumes from 0 to 8 ml, by 0.5-ml steps from 0 to 3 ml and 1-ml steps from 3 to 8 ml. At each volume step, the balloon was completely deflated by applying a negative pressure, then fully inflated with 10 ml of air and finally deflated to the target volume. Static pressures were obtained by airway occlusion maneuvers of 5 s. From those data, we obtained two curves expressing the individual pressure-volume (PV) relationship between balloon filling volume and Pes, respectively at end-expiration and end-inspiration (Fig. 1).

On the end-expiratory PV curve we graphically identified the intermediate linear section and its lower and upper limits, expressed as minimum and maximum filling volumes (V_MIN_ and V_MAX_). According to in vitro results [8], V_MIN_ is the smaller filling volume to be injected into the esophageal catheter to pressurize it at the same level of the pressure surrounding the esophageal balloon, i.e., to reach a balloon transmural pressure of zero. Further volume injection induces progressive inflation of the esophageal balloon, V_MAX_ being the larger filling volume that does not induce overstretch of the balloon wall [8]. Therefore, the range between V_MIN_ and V_MAX_ was considered to correspond to appropriate balloon filling, with volumes below V_MIN_ denoting underfilling and volumes above V_MAX_ denoting overfilling of the balloon. Within the appropriate volume range, we identified the volume providing the maximum difference between Pes_EI_ and Pes_EE_ (V_BEST_).

The slope of the intermediate linear section of the end-expiratory PV curve was obtained by least square fitting and it was considered to express the elastance of the esophagus (Ees) [7]. For any filling volume V_X_ above V_MIN_, the pressure generated by the esophageal wall (Pew) was calculated as: Pew = (V_X_ – V_MIN_) * Ees (Figure S1 in Additional file 1). Pew was considered null at filling volumes lower than V_MIN_, the balloon transmural pressure being negative and therefore the esophagus reaction to balloon filling negligible in this case.

**Fig. 2:**
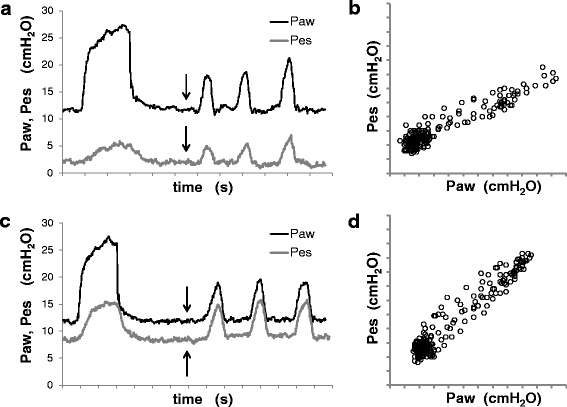
Validation test at different esophageal catheter-filling volumes. Panels (**a**) and (**c**): Paw and Pes over time, during a single mechanical respiratory breath and an end-expiratory occlusion maneuver with chest compressions. *Arrows* refer to the start of the occlusion maneuver. Panels (**b**) and (**d**): Pes/Paw relative changes during the end-expiratory occlusion maneuver. Panels (**a**) and (**b**) correspond to an esophageal balloon catheter filling of 0.5 ml, while panels (**c**) and (**d**): correspond to a filling of 2.5 ml. The slope of the Pes/Paw relationship was 0.48 with an injected volume of 0.5 ml (panel **b**) and 1.02 with an injected volume of 2.5 ml (panel **d**). *Paw* airway pressure, *Pes* esophageal pressure

Then we compared five different balloon filling strategies: 0.5 ml (V_0.5_), as per traditional recommendations; 4.0 ml (V_4.0_), as per manufacturer’s recommendations; 8.0 ml (V_8.0_), i.e., approximately at full balloon inflation; filling volume equal to individual V_MIN_; and filling volume equal to individual V_BEST_. At each of these filling volumes, we measured again the static Pes_EE_ and Pes_EI_ and performed a validation occlusion test [3, 11]. Our patients being sedated and paralyzed, we recorded simultaneous changes of Pes (ΔPes) and airway pressure (Paw) (ΔPaw) while applying gentle compressions on the patient’s chest during an end-expiratory occlusion maneuver (Fig. 2). The hypothesis that V_BEST_ was associated with the ΔPes/ΔPaw ratio closest to 1 among the different balloon filling strategies was evaluated by repeated measures ANOVA and Cochran’s Q test.

From the Pes measurements obtained at a filling volume equal to individual V_BEST_ and the corresponding Pew, we computed the calibrated values of Pes (Pes_CAL_), as previously suggested [6, 7]: Pes_CAL_ = Pes – Pew. The hypothesis that Pes_CAL_ significantly differed from raw measurements of Pes at V_0.5_ (Pes_V0.5_), V_4.0_ (Pes_V4.0_) and V_BEST_ (Pes_VBEST_) was evaluated by Bland-Altman analysis and repeated measures ANOVA. The study was approved by the committee on research ethics at the institution in which the enrollment was conducted (Fondazione IRCCS Policlinico S. Matteo, Pavia, Italy) and informed consent was obtained as required.

## Results

Fifty series of measurements were performed in 36 patients (20 males, 57 ± 17 years) undergoing pressure controlled mechanical ventilation. In nine patients, multiple measurements at different body positions and/or positive end-expiratory pressure (PEEP) settings were performed: six patients were studied twice, one patient three times and two patients four times. Body position was supine with the bed at 20–30 degrees head up, lateral and prone in 45, 3 and 2 cases respectively. Settings of pressure-controlled mechanical ventilation were: inspiratory oxygen fraction (FiO_2_) 0.71 ± 0.22, PEEP 11.8 ± 5.0 cmH_2_O, peak pressure 28.6 ± 5.5 cmH_2_O, respiratory rate 15.7 ± 5.2 bpm. Tidal volume/ideal body weight was 7.9 ± 1.8 ml/kg, plateau pressure 27.6 ± 5.0 cmH_2_O and partial pressure of oxygen in arterial blood (PaO_2_)/FiO_2_ ratio 165 ± 76 mmHg. Causes of acute respiratory failure were: community-acquired pneumonia (7), heart failure (2), chronic obstructive pulmonary disease (COPD) decompensation (1), acute respiratory distress syndrome (ARDS) (7), abdominal compartment syndrome (6), pulmonary alveolar proteinosis (8), and postoperative respiratory complications (5). Pulmonary alveolar proteinosis patients were studied during the whole lung lavage procedure previously described [12]. In five patients, body mass index was higher than 50 kg/m^2^.

### Esophageal balloon pressure-volume curves in mechanically ventilated patients

End-expiratory and end-inspiratory complete esophageal balloon PV curves were obtained in all cases; in some patient, filling volumes larger than 6 ml stimulated esophageal peristaltic contractions that were rapidly self-limiting. An intermediate linear section of the end-expiratory esophageal balloon PV curve was graphically detected in all cases. Lower and upper limit of this section, i.e., V_MIN_ and V_MAX_, were 1.5 ± 0.6 and 5.4 ± 0.9 cmH_2_O, respectively. V_MIN_ ranged from 0.0 to 3.0 ml and its value directly correlated with Pes_EE_ (r = 0.766, 95 % CI 0.620 to 0.861; *p* < 0.0001). Mean value of V_BEST_ was 3.5 ± 1.9 ml (range 0.5–6.0). V_BEST_ directly correlated with Pes_EI_ (r = 0.528, 95 % CI 0.292 to 0.703; *p* < 0.0001). The slope of the linear section of the curve, i.e., the esophageal elastance (Ees), was 1.1 ± 0.5 cmH_2_O/ml (range 0.3–3.1). Ees was higher in females than in males (1.3 ± 0.6 vs. 1.0 ± 0.4 cmH_2_O/ml; *p* = 0.04) and was negatively correlated with body mass index (r = -0.629, 95 % CI -0.772 to -0.426; *p* < 0.0001).

**Fig. 3:**
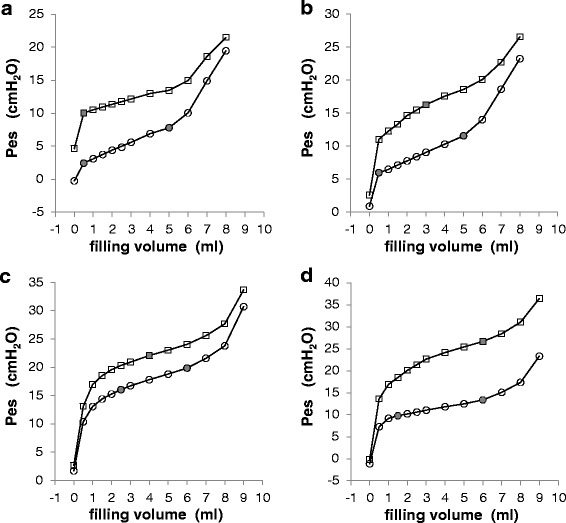
Examples of inspiratory and expiratory static esophageal balloon pressure-volumes curves. *Circles* refer to end-expiratory esophageal pressure (Pes_EE_); *closed circles* refer to V_MIN_ and V_MAX_ as graphically detected (respectively lower and upper limits of the intermediate linear section of the curve). *Squares* refer to end-inspiratory esophageal pressure (Pes_EI_); *closed squares* refer to V_BEST_ (the filling volume associated with the largest Pes_EI_ – Pes_EE_ difference). Panel (**a**) 32-year-old male patient, body mass index (BMI) 27 kg/m^2^; pulmonary alveolar proteinosis and kyphoscoliosis; positive end-expiratory pressure (PEEP) 0 cmH_2_0; tidal volume (TV) 550 ml, plateau pressure (Pplat) 20 cmH_2_O. V_MIN_ and V_BEST_ 0.5 ml. Esophageal elastance 1.3 cmH_2_O/ml, pressure generated by the esophageal wall (Pew) at V_BEST_ 0.0 cmH_2_O. Panel (**b**) 82-year-old female patient, BMI 22 kg/m^2^; respiratory failure after pulmonary endoarterectomy; PEEP 7 cmH_2_0, TV 500 ml, Pplat 25 cmH_2_O. V_MIN_ 0.5 ml and V_BEST_ 3 ml. Esophageal elastance 1.3 cmH_2_O/ml, Pew at V_BEST_ 3.3 cmH_2_O. Panel (**c**) 31-year-old male patient, BMI 63 kg/m^2^; legionella pneumonia and morbid obesity; PEEP 12 cmH_2_0, TV 450 ml, Pplat 30 cmH_2_O. V_MIN_ 2.5 ml and V_BEST_ 4.0 ml. Esophageal elastance 1.2 cmH_2_O/ml, Pew at V_BEST_ 1.8 cmH_2_O. Panel (**d**) 70-year-old male patient, BMI 23 kg/m^2^; intra-abdominal hypertension due to large retroperitoneal hematoma; PEEP 10 cmH_2_0, TV 370 ml, Pplat 29 cmH_2_O. V_MIN_ 1.5 ml and V_BEST_ 6 ml. Esophageal elastance 0.8 cmH_2_O/ml, Pew at V_BEST_ 3.6 cmH_2_O. *Pes* esophageal pressure

Figure 3 shows representative end-expiratory and end-inspiratory static esophageal balloon pressure-volume curves obtained in four different patients. Figure S2 in Additional file 1 shows the average end-expiratory and end-inspiratory esophageal balloon pressure-volume curves, and Figure S3 in Additional file 1 shows three end-expiratory static esophageal balloon pressure-volume curves obtained at three different PEEP levels in the same patient.

### Effects of balloon inflation on pressure transmission and esophageal wall pressure

**Fig. 4:**
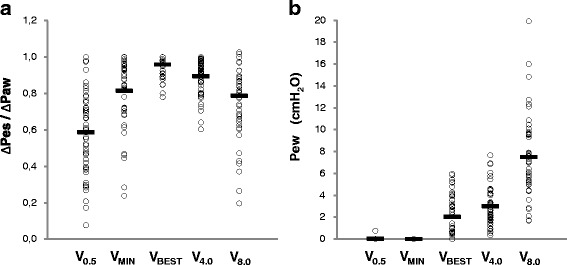
Effects of different esophageal balloon filling volumes on the validation test and the esophagus artifact. Panel (**a**) The validation occlusion test performed at V_BEST_ was associated with the ∆Pes/∆Paw ratio closest to 1 (0.96 ± 0.06; *p* < 0.0001 compared to all the other filling strategies) and the highest success rate (98 %; *p* < 0.001 compared to all the other filling strategies). Panel (**b**) Pressure generated by the esophageal wall (Pew) as a reaction to optimal filling volume (V_BEST_) was 2.0 ± 1.9 cmH_2_O and ranged from 0.0 to 6.0 cmH_2_O. Pew measured at lower filling volumes (V_0.5_ and V_MIN_) was lower (*p* < 0.0001) and Pew measured at near-full balloon inflation (V_8.0_) was higher (*p* < 0.0001). *Open symbols* refer to individual data; *bars* refer to mean values. ∆*Pes/*∆*Paw* ratio between esophageal pressure (Pes) and airway pressure (Paw) changes during the validation occlusion test

Pes_EE_ and Pes_EI_ progressively increased with the increase of balloon filling volume (*p* < 0.0001). ∆Pes was 5.3 ± 2.3 cmH_2_O at V_BEST_ and it was decreased by 7 %, 17 %, 17 %, and 39 % when the balloon was filled at V_4.0_, V_MIN_, V_8.0_, and V_0.5_ respectively (*p* < 0.0001). The ∆Pes/ΔPaw ratio closest to 1 was observed at V_BEST_ (0.96 ± 0.06; *p* < 0.0001 vs. all the other filling strategies). ∆Pes/ΔPaw ratio was in the 0.8–1.2 range in 98 %, 88 %, 64 %, 60 %, and 22 % of cases when the balloon was inflated at V_BEST_, V_4.0_, V_MIN_, V_8.0_, and V_0.5_ respectively (*p* < 0.001). At V_BEST_, Pew was 2.0 ± 1.9 cmH_2_O (range 0.0–6.0). These findings are summarized in Table 1 and Fig. 4.

**Table 1:** Esophageal pressure measurements in patients under positive-pressure ventilation with five different esophageal balloon filling strategies

	Volume (ml)	Pes_EE_ (cmH_2_O)	Pes_EI_ (cmH_2_O)	∆Pes (cmH_2_O)	∆Pes/∆Paw	Pew (cmH_2_O)
V_0.5_	0.5 ± 0.0^*^	7.3 ± 3.6^*^	10.6 ± 3.8^*^	3.3 ± 1.9^£^	0.59 ± 0.23^£^	0.0 ± 0.1^^^
V_MIN_	1.5 ± 0.6^*^	10.4 ± 5.2^*^	14.8 ± 5.7^*^	4.4 ± 2.0^$^	0.81 ± 0.19^$^	0.0 ± 0.0^^^
V_BEST_	3.5 ± 1.9^#^	12.5 ± 5.2^#^	17.8 ± 5.6^#^	5.3 ± 2.3^*^	0.96 ± 0.06^*^	2.0 ± 1.9^#^
V_4.0_	4.0 ± 0.0^#^	13.4 ± 4.4^#^	18.4 ± 4.7^#^	4.9 ± 2.1^§^	0.89 ± 0.10^§^	3.0 ± 1.7^#^
V_8.0_	8.0 ± 0.0^*^	23.8 ± 6.5^*^	28.2 ± 6.1^*^	4.4 ± 2.4^$^	0.79 ± 0.21^$^	7.5 ± 3.6^*^

*Pes*_*EE*_ *, Pes*_*EI*_ *and *∆*Pes* end-expiratory, end-inspiratory, and tidal swing of Pes respectively, *∆Pes/∆Paw* esophageal to airway pressure change ratio during an occlusion test (airway opening occlusion and chest compressions), *Pew* pressure generated by the esophageal wall as a reaction to balloon volume increase, *V*_*0.5*_ *, V*_*4.0*_ *and V*_*8.0*_ injected volumes of 0.5, 4, and 8 ml respectively, *V*_*MIN*_ lower limit of the linear section of the end-expiratory PV curve, *V*_*BEST*_ injected volume associated with the largest Pes swing (see Methods and Fig. 1 for details)

^*^ *p* < 0.0001 vs. all the other injected volumes; ^#^ *p* < 0.0001 vs. V_0.5_, V_MIN_, and V_8.0_; ^^^ *p* < 0.0001 vs. V_4.0_, V_8.0_, and V_BEST_; ^§^ *p* < 0.0001 vs. V_0.5_ and V_BEST_, *p* < 0.05 vs. V_8.0_ and V_MIN_; ^$^ *p* < 0.0001 vs. V_0.5_ and V_BEST_, *p* < 0.05 vs. V_4.0_; ^£^ *p* < 0.0001 vs. V_MIN_, V_BEST_, and V_4.0_, *p* < 0.05 vs. V_8.0_

### Calibration procedure

**Fig. 5:**
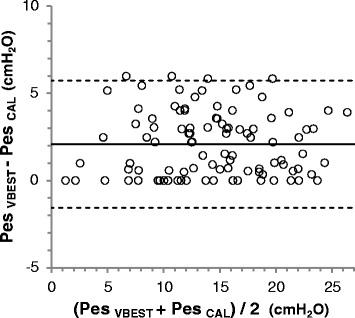
Subtraction of the esophageal artifact: effect on absolute values of Pes. Compared to Pes_CAL_, bias (mean difference, *continuous line*) and precision (±1.96 SD of the difference, *dotted lines*) of Pes_VBEST_ were 2.1 ± 3.6 cmH_2_O. In individual patients, overestimation of Pes [and underestimation of transpulmonary pressure (P_L_)] due to esophageal elastance may be clinically significant, eventually leading to inappropriate high positive end-expiratory pressure (PEEP) levels and/or end-inspiratory lung overdistention. *Pes* esophageal pressure

Considering end-expiratory and end-inspiratory conditions all together, mean value of Pes_CAL_ was 13.0 ± 5.9 cmH_2_O; compared to Pes_CAL_, Pes_V0.5_ was significantly lower (9.0 ± 4.0 cmH_2_O; *p* < 0.0001), whereas Pes_V4.0_ (15.9 ± 5.1 cmH_2_O; *p* < 0.0001) and Pes_VBEST_ (15.1 ± 6.0 cmH_2_O; *p* < 0.0001) were significantly higher. Bland-Altman analyses are shown in Fig. 5 and in Figure S4 of Additional file 1.

## Discussion

We evaluated esophageal manometry in patients with ARF under positive-pressure mechanical ventilation. Our main findings are:

1. The optimal filling volume of the esophageal balloon (V_BEST_) can be easily identified as the one that maximizes respiratory ΔPes.
2. The value of V_BEST_ varies in a large range, typically is much larger than traditionally used small filling volumes, and thus is frequently associated with a significant esophageal wall pressure (Pew).
3. A simple calibration procedure, based on balloon filling at V_BEST_ and subtracting the associated Pew, allows improvement of the assessment of both relative changes and absolute values of Pes.

Esophageal pressure is used as surrogate for pleural pressure (Ppl) in order to compute transpulmonary (airways minus pleural) pressure (P_L_), i.e., the effective pressure distending the lung parenchyma. Ideally, in mechanically ventilated patients we should maintain positive values of P_L_ at end-expiration while avoiding excessive P_L_ at end-inspiration, to limit both lung derecruitment and overdistention. These theoretical assumptions were recently translated into two different practical approaches to set PEEP, with potential advantages in ARF [1, 2]. The two approaches are based on different methods to compute transpulmonary pressure: Talmor and colleagues assumed the absolute value of esophageal pressure to be equal to pleural pressure (direct method), whereas Grasso and colleagues computed pleural pressure by partitioning airway pressure according to respiratory swings of esophageal pressure (elastance-derived method).

It was recently demonstrated that, under positive-pressure conditions, the use of traditional small filling volumes is associated with underfilling and thus malfunctioning of the esophageal balloon catheter in most cases [8]. Underfilling involves under-transmission of both absolute values and respiratory swings of esophageal pressure, and therefore may affect both the direct and the elastance-derived method.

In this study in patients with ARF under controlled mechanical ventilation, the minimum filling volume (V_MIN_) that was able to accurately transmit the end-expiratory esophageal pressure was substantially larger than 0.5 ml in most cases, being on average 1.5 ml and reaching values as high as 3 ml. As previously observed in vitro [8], the in vivo V_MIN_ was proportional to the surrounding pressure: the higher the Pes, the larger the volume to be injected into the catheter. Moreover, the filling volume providing optimal transmission of Pes respiratory swings (V_BEST_) was substantially larger than V_MIN_, being on average 3.5 ml and directly related to the Pes value. The positive relationship between V_BEST_ and Pes is explained by the fact that part of the volume injected into the esophageal catheter must be spent to pressurize the system at the pressure level surrounding the balloon. The dynamic occlusion test is the classic method to validate a Pes measurement [3, 11]: similar Pes and Paw swings should be observed during spontaneous respiratory efforts (or manual chest compressions in the case of a passive patient) against the closed airway opening. A major finding of our study is that in almost all cases the balloon filling volume required to pass the validation occlusion test was much larger than 0.5 ml. Therefore we suggest abandoning the systematic use of the “traditional” small filling volumes. A simple option could be to use a fixed higher filling volume. However, V_BEST_ was highly variable among different patients and conditions, ranging from 0.5 to 6 ml in our series. This suggests that the esophageal catheter filling volume should be adapted to the intrathoracic pressure condition of any given patient. Therefore, in order to obtain reliable esophageal pressure waveforms, we suggest filling the esophageal balloon catheter progressively step-by-step and selecting, within the catheter-specific range of filling volumes [8], the volume associated with the largest esophageal pressure tidal swing. Since dynamic and static tidal variation of Pes are very similar [13], for clinical purposes the procedure can be performed without occlusion maneuvers at each filling step. Many factors, like mechanical ventilation setting, body position, fluid balance and intra-abdominal pressure, can influence intrathoracic pressure in critically ill patients. At any significant change of one or more of these factors, for better results the optimal filling volume of the esophageal balloon catheter should be rechecked.

The use of larger filling volumes stimulated significant esophageal reaction in our patients. The pressure generated by the esophageal wall, when progressively distended, has both a passive (collagen and elastic fibers) and an active (smooth muscle tone) component, as well described by Orvar and colleagues [14]. When a balloon was progressively inflated in the esophagus of awake healthy volunteers, the pressure generated by the esophageal wall (Pew) was linearly related to the esophageal cross-sectional area (CSA): for each 10 mm^2^ increase in CSA above 50 mm^2^, Pew increased by 1 cmH_2_O. Thresholds for peristaltic contractions and for pain or pressure sensation were 150 and 300 mm^2^ respectively. The esophageal catheter we used is a nasogastric tube (20 mm^2^ CSA) provided with a 10-cm-long esophageal balloon that reaches 180 mm^2^ CSA when fully inflated with approximately 10 ml. Therefore, a detectable Pew was expected at intermediate balloon filling volumes (but not in case of deflated or minimally inflated balloon), whereas peristaltic contractions were expected only for higher filling volumes, approximating full balloon inflation. On average we observed a Pew of about 1 cmH_2_O for each milliliter of injected volume above V_MIN_. When V_BEST_ was injected into the catheter, Pew was 2 cmH_2_O on average and reached values as high as 6 cmH_2_O. With a balloon near fully inflated, Pew was about 8 cmH_2_O on average with single values as high as 20 cmH_2_O. This esophageal reaction to balloon inflation is a well-known artifact affecting absolute values of esophageal pressure, which may therefore become substantially more positive than pleural pressure [5, 7]. Such an artifact can result in significant and unpredictable overestimation of Pes and underestimation of P_L_, possibly leading to inappropriately high PEEP levels and/or end-inspiratory lung overdistention when a Pes-guided mechanical ventilation strategy is adopted. Our data suggest that the risk overestimation of Pes is higher in case of female subjects and/or moderate-low body mass indexes. These findings are consistent with gender differences in the responses to esophageal balloon distention [15] and high prevalence of hypomotility of the esophageal body in obese patients [16]. Milic Emili and colleagues [6] suggested using very small filling volumes for the esophageal balloon in order to easily minimize the esophageal artifact and their recommendation survived for about 50 years, being only recently rediscussed [3, 8]. Our findings prove that, in mechanically ventilated ARF patients, it is not possible to avoid balloon underfilling and esophageal artifact at the same time. This technical dilemma may explain why different research groups obtained low [17], moderate [18, 19] or high [9] absolute Pes values in the same condition of supine subjects at functional residual capacity.

To solve this technical problem, we reconsidered and adapted a procedure originally proposed by Milic-Emili who suggested – as an alternative to small filling volumes – to extrapolate to zero balloon volume the Pes measured at higher filling volumes [6]. Our calibration procedure significantly improved the quality of Pes measurement. Tidal swings of Pes were maximized, thus allowing passing of the validation test in almost all cases. Moreover, it was possible to recognize and subtract from Pes the pressure generated by the esophageal wall, which would have significantly raised Pes above the pleural value. In our opinion, calibrated Pes represents the best surrogate for absolute pleural pressure in critical care patients and may significantly improve the direct method proposed by Talmor and colleagues [1].

### Limitations of the study

Since we did not obtain direct measurements of pleural pressure in our patients, our statement that calibrated Pes reflects Ppl better than uncalibrated Pes is based on the reasonable assumption that the removal of artifacts should improve a measure. Our calibration procedure was designed according to general principles and therefore for application to different esophageal catheters. However, the study was performed with a single specific esophageal balloon catheter and therefore the results cannot be straightly generalized. Esophageal balloon catheters of different manufacturers show a very similar behavior when progressively inflated under positive-pressure conditions, the major difference among devices being the amplitude of appropriate filling volumes range [8]. The catheter used in the present study is a nasogastric tube provided with a large esophageal balloon with a wide range of appropriate filling volumes. With different esophageal catheters we should expect different optimal filling volumes, different esophageal reaction, and therefore different calibration factors. We studied passive patients under controlled mechanical ventilation: the feasibility of our calibration procedure in active patients should be verified. In particular, during assisted spontaneous breathing, the variability of end-expiratory lung volume might prevent the correct computation of esophageal elastance, while the variability of tidal volume might make detection of optimal filling volume more difficult.

## Conclusions

The present study demonstrates that an approach based on a calibrated esophageal pressure is feasible and provides significant improvement of the technique over traditional approaches. Clinical advantages of using a calibrated esophageal pressure to guide mechanical ventilation should be demonstrated by future trials.

## Key messages

- Esophageal pressure measurement can be affected by inappropriate balloon filling and esophageal elastance.
- In mechanically ventilated patients, traditional small filling volumes are almost always inappropriate.
- Optimal filling volume within the catheter-specific appropriate range is easy to find as the one that maximizes respiratory ΔPes.
- The pressure generated by the esophagus wall as a reaction to optimal balloon filling may significantly affect Pes.
- In vivo calibration of Pes, by selecting the optimal balloon filling and subtracting the esophagus artifact, makes Pes-guided mechanical ventilation more reliable.

## Additional file

Additional file 1
**Figure S1.** In vitro and in vivo Pressure-Volume curves of the esophageal balloon. Within the intermediate linear section of the esophageal balloon PV curve, Pes is stable when the balloon is inflated in vitro (dotted line), whereas Pes linearly increases with the increase of the filling volume in vivo (open circles) due to esophageal elastance (Ees). When pressure generated by the esophageal wall is subtracted from Pes (Pes-Pew; closed circles), in vitro and in vivo curves closely parallel each other. Dotted line: in vitro PV curve obtained by progressive inflation of the balloon while the surrounding pressure was maintained at 10 cmH_2_O. Open circles: end-expiratory PV curve obtained in a patient under controlled mechanical ventilation with high positive end-expiratory pressure (15 cmH_2_O). Closed circles: end-expiratory PV curve obtained by subtraction of Pew from raw Pes values in the same patient. **Figure S2.** Average end-expiratory and end-inspiratory esophageal balloon pressure-volume curves in acute respiratory failure patients. Open circles: end-expiratory static Pes values (Pes_EE_); open squares: end-inspiratory static Pes values (Pes_EI_). Vertical lines refer to standard deviation of Pes. Horizontal lines refer to standard deviation of specific filling volumes detected on the esophageal balloon pressure-volume curves (V_MIN_, V_BEST_ and V_MAX_). V0.5 was lower than V_MIN_, i.e. it was associated to balloon underfilling at end-expiration, in 42 cases (84 %); V_0.5_ was lower than V_BEST_, i.e. it was associated to suboptimal ΔPes in 47 cases (94 %). V_MIN_ was lower than V_BEST_ in 37 cases (74 %), being the V_BEST_ - V_MIN_ difference 2.0 ± 1.7 ml. **Figure S3.** End-expiratory static esophageal balloon pressure-volume curves at different PEEP levels. In panel A, raw Pes values are presented; in panel B, the pressure generated by the esophageal wall (Pew) is subtracted from Pes value. Circles: ZEEP; squares: PEEP 5 cmH_2_O; triangles: PEEP 15 cmH_2_O. Closed symbols refer to V_BEST_, i.e. the balloon filling volume corresponding to the largest respiratory ΔPes (not displayed in figure). In the same patient, at increasing level of PEEP, a progressively larger balloon filling volume is needed to optimize respiratory ΔPes. Optimal filling volume (V_BEST_) stimulates a variable esophageal pressure reaction, confounding Pes measurement. For example, by filling the esophageal balloon with 5 ml, corresponding to V_BEST_ at PEEP 15 cmH_2_O, raw Pes values at the three PEEP levels are very similar (panel A). Once the pressure generated by the esophageal wall is subtracted from Pes values, the PEEP-induced increase of the pressure surrounding the esophagus becomes clearly detectable (Panel B). **Figure S4.** Pes measured with traditional low filling volume (V0.5) or with manufacturer’s recommended filling volume (V4.0) compared to calibrated Pes. Panel A. Compared to calibrated Pes, bias and precision (± 1.96 SD) of Pes_V0.5_ were -4.1 ± 5.5 cmH_2_O. The Pes_0.5_ - PesCAL difference inversely correlated with the Pes_0.5_ - Pes_CAL_ mean value (R= -0.694, p<0.0001): the higher the Pes value, the higher the Pes underestimation due to balloon underfilling. Panel B. Compared to calibrated Pes, bias and precision (± 1.96 SD) of Pes_V4.0_ were -2.9 ± 3.3 cmH_2_O. The Pes_4.0_ - Pes_CAL_ difference inversely correlated with the Pes_4.0_ - PesCAL mean value (R= -0.470, p<0.0001): the lower the Pes value, the higher the Pes overestimation due to the esophageal reaction to balloon inflation at 4 ml. (PDF 424 kb)

## Abbreviations

∆Pes: respiratory increase of static Pes (Pes_EI_ – Pes_EE_)
∆Pes/∆Paw: ratio between Pes and Paw changes during the validation occlusion test
ARF: acute respiratory failure
CSA: cross-sectional area
Ees: esophagus elastance
FiO_2_: inspiratory oxygen fraction
Paw: airway pressure
PEEP: positive end-expiratory pressure
Pes: esophageal pressure
Pes_CAL_: calibrated value of Pes
Pes_EE_ and Pes_EI_: static end-expiratory and end-inspiratory Pes
Pes_V0.5_, Pes_V4.0_, Pes_V8.0_, Pes_VMIN_, Pes_VBEST_: Pes measured with esophageal catheter filling volumes of 0.5 ml, 4 ml, 8 ml, V_MIN_ and V_BEST_ respectively
Pew: pressure generated by the esophagus wall as a reaction to balloon filling
P_L_: transpulmonary pressure
Ppl: pleural pressure
PV curve: pressure-volume curve
V_0.5_, V_4.0_ and V_8.0_: injected volumes of 0.5, 4 and 8 ml respectively
V_BEST_: injected volume associated with the largest ∆Pes
V_MIN_ and V_MAX_: injected volumes corresponding to the lower and the upper limits of the linear section of the end-expiratory esophageal balloon PV curve
