# Supplementary material for: In vivo calibration of esophageal pressure in the mechanically ventilated patient makes measurements reliable
Source: Crit Care. 2016 Apr 11;20:98. doi: 10.1186/s13054-016-1278-5 (PMC4827205; doi:10.1186/s13054-016-1278-5)
Supplement: Additional file 1 — Figure S1. In vitro and in vivo Pressure-Volume curves of the esophageal balloon. Within the intermediate linear section of the esophageal balloon PV curve, Pes is stable when the balloon is inflated in vitro (dotted line), whereas Pes linearly increases with the increase of the filling volume in vivo (open circles) due to esophageal elastance (Ees). When pressure generated by the esophageal wall is subtracted from Pes (Pes-Pew; closed circles), in vitro and in vivo curves closely parallel each other. Dotted line: in vitro PV curve obtained by progressive inflation of the balloon while the surrounding pressure was maintained at 10 cmH2O. Open circles: end-expiratory PV curve obtained in a patient under controlled mechanical ventilation with high positive end-expiratory pressure (15 cmH2O). Closed circles: end-expiratory PV curve obtained by subtraction of Pew from raw Pes values in the same patient. Figure S2. Average end-expiratory and end-inspiratory esophageal balloon pressure-volume curves in acute respiratory failure patients. Open circles: end-expiratory static Pes values (PesEE); open squares: end-inspiratory static Pes values (PesEI). Vertical lines refer to standard deviation of Pes. Horizontal lines refer to standard deviation of specific filling volumes detected on the esophageal balloon pressure-volume curves (VMIN, VBEST and VMAX). V0.5 was lower than VMIN, i.e. it was associated to balloon underfilling at end-expiration, in 42 cases (84 %); V0.5 was lower than VBEST, i.e. it was associated to suboptimal ΔPes in 47 cases (94 %). VMIN was lower than VBEST in 37 cases (74 %), being the VBEST - VMIN difference 2.0 ± 1.7 ml. Figure S3. End-expiratory static esophageal balloon pressure-volume curves at different PEEP levels. In panel A, raw Pes values are presented; in panel B, the pressure generated by the esophageal wall (Pew) is subtracted from Pes value. Circles: ZEEP; squares: PEEP 5 cmH2O; triangles: PEEP 15 cmH2O. Closed symbols refer to VBEST, i.e. t [file 13054_2016_1278_MOESM1_ESM.pdf]

In vivo calibration of esophageal pressure in the mechanically ventilated patient makes measurements reliable – Supplementary material

Mojoli F [1,2], Iotti GA [1], Torriglia F [2], Pozzi M [1], Volta CA [3], Bianzina S [2], Braschi A [1,2], Brochard L [4,5]

[1] Anesthesia and Intensive Care, Emergency Department, Fondazione IRCCS Policlinico S. Matteo, Pavia, Italy

[2] Anesthesia, Intensive Care and Pain Therapy, Department of Clinical, Surgical, Diagnostic and Pediatric Sciences, University of Pavia, Pavia, Italy

[3] Anesthesia and Intensive Care, Department of Morfology, Surgery and Experimental Medicine, University of Ferrara, Arcispedale Sant'Anna, Ferrara, Italy

[4] Keenan Research Centre, Li Ka Shing Knowledge Institute, St. Michael's Hospital, Toronto, Ontario, Canada

[5] Interdepartmental Division of Critical Care Medicine, University of Toronto, Toronto, Ontario, Canada.

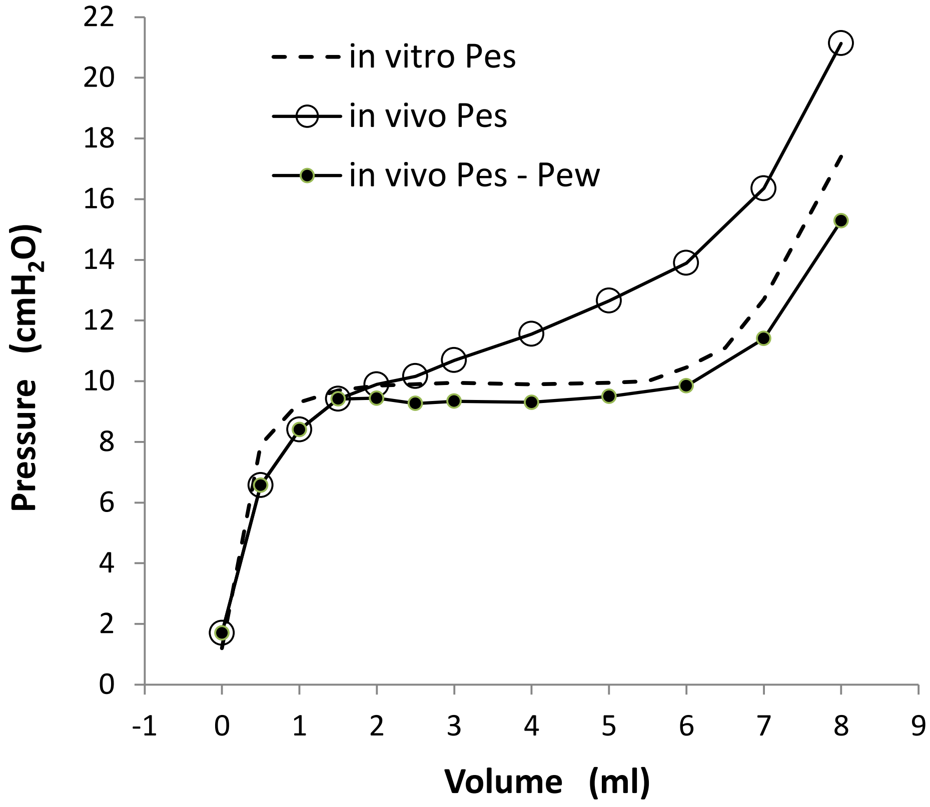

Figure S1. ***In vitro*** **and** ***in vivo*** **Pressure-Volume curves of the esophageal balloon.**

Within the intermediate linear section of the esophageal balloon PV curve, Pes is stable when the balloon is inflated in vitro (dotted line), whereas Pes linearly increases with the increase of the filling volume i*n vivo* (open circles) due to esophageal elastance (Ees). When pressure generated by the esophageal wall is subtracted from Pes (Pes-Pew; closed circles), in vitro and in vivo curves closely parallel each other.

Dotted line: in vitro PV curve obtained by progressive inflation of the balloon while the surrounding pressure was maintained at 10 cmH_2O. Open circles: end-expiratory PV curve obtained in a patient under controlled mechanical ventilation with high positive end-expiratory pressure (15 cmH_2O). Closed circles: end-expiratory PV curve obtained by subtraction of Pew from raw Pes values in the same patient.

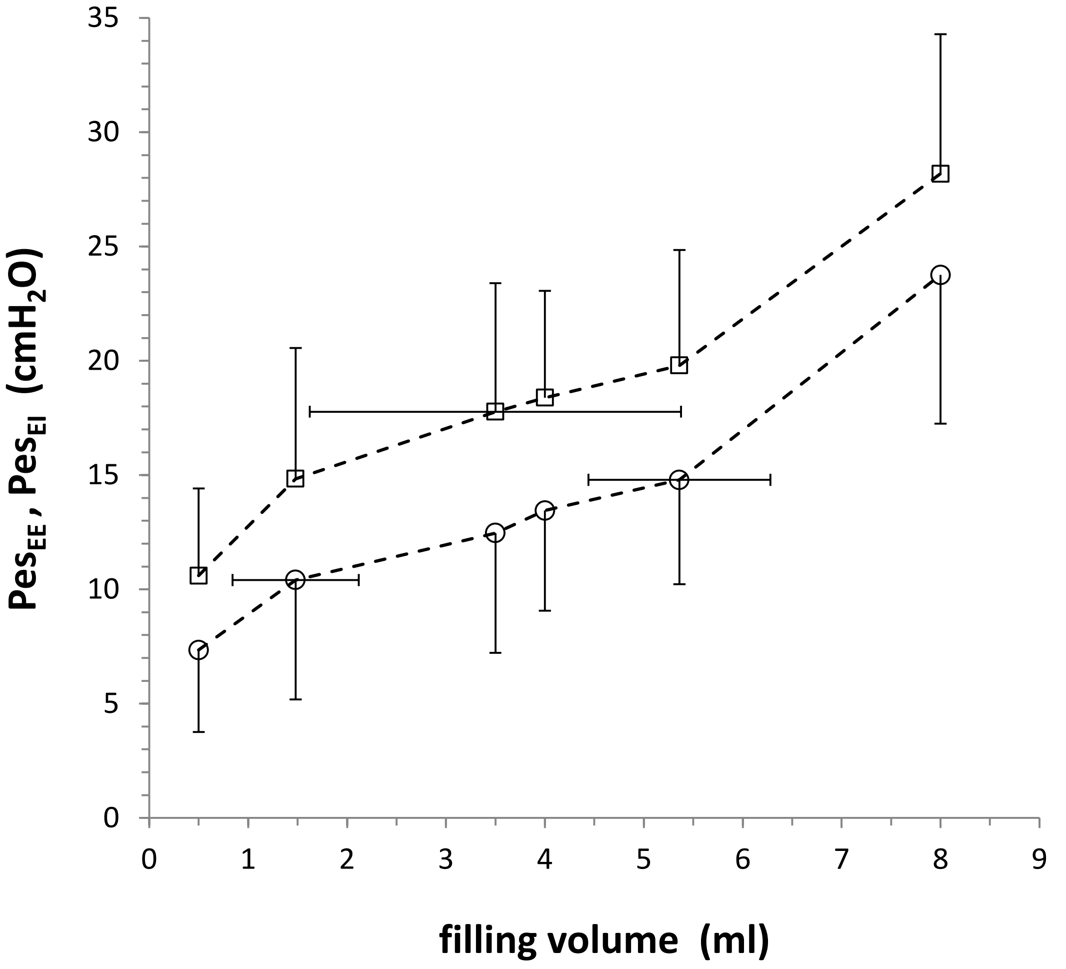

Figure S2. Average end-expiratory and end-inspiratory esophageal balloon pressure-volume curves in acute respiratory failure patients.

Open circles: end-expiratory static Pes values (Pes_{EE}); open squares: end-inspiratory static Pes values (Pes_{EI}). Vertical lines refer to standard deviation of Pes. Horizontal lines refer to standard deviation of specific filling volumes detected on the esophageal balloon pressure-volume curves (V_{MIN}, V_{BEST} and V_{MAX}).

$V_{0.5}$ was lower than V_{MIN}, i.e. it was associated to balloon underfilling at end-expiration, in 42 cases (84%); $V_{0.5}$ was lower than V_{BEST}, i.e. it was associated to suboptimal ΔPes in 47 cases (94%). V_{MIN} was lower than V_{BEST} in 37 cases (74%), being the V_{BEST} - V_{MIN} difference 2.0 ± 1.7 ml.

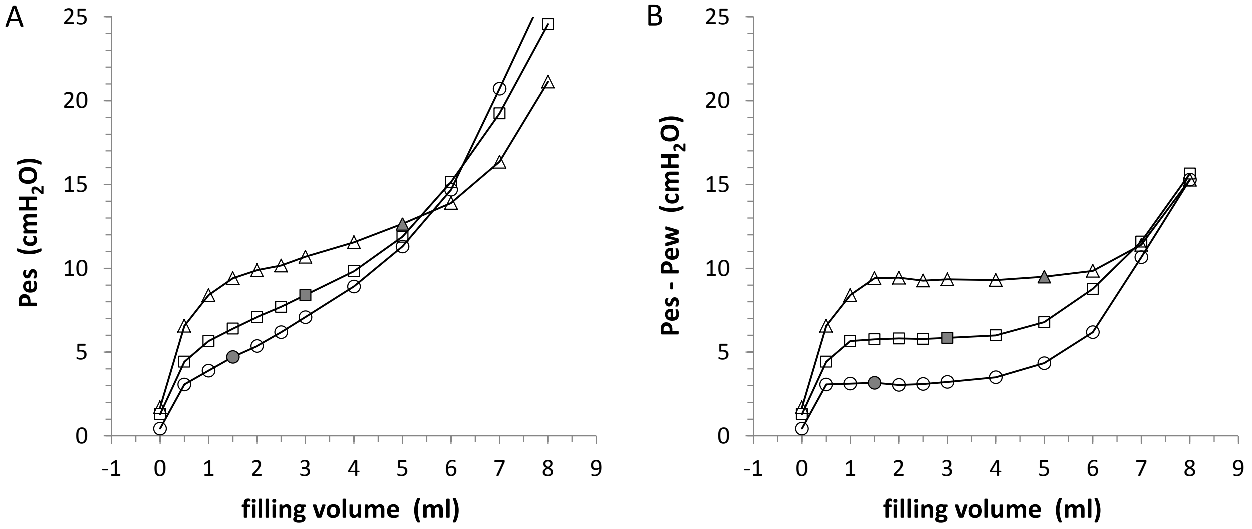

Figure S3. End-expiratory static esophageal balloon pressure-volume curves at different PEEP levels.

In panel A, raw Pes values are presented; in panel B, the pressure generated by the esophageal wall (Pew) is subtracted from Pes value. Circles: ZEEP; squares: PEEP 5 cmH_2O; triangles: PEEP 15 cmH_2O. Closed symbols refer to V_{BEST}, i.e. the balloon filling volume corresponding to the largest respiratory ΔPes (not displayed in figure).

In the same patient, at increasing level of PEEP, a progressively larger balloon filling volume is needed to optimize respiratory ΔPes. Optimal filling volume (V_{BEST}) stimulates a variable esophageal pressure reaction, confounding Pes measurement. For example, by filling the esophageal balloon with 5 ml, corresponding to V_{BEST} at PEEP 15 cmH_2O, raw Pes values at the three PEEP levels are very similar (panel A). Once the pressure generated by the esophageal wall is subtracted from Pes values, the PEEP-induced increase of the pressure surrounding the esophagus becomes clearly detectable (Panel B).

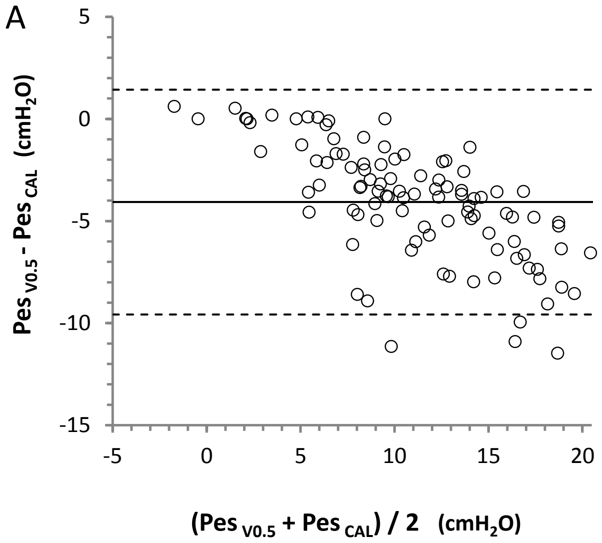

Figure S4. Pes measured with traditional low filling volume ($V_{0.5}$) or with manufacturer's recommended filling volume ($V_{4.0}$) compared to calibrated Pes.

Panel A. Compared to calibrated Pes, bias and precision (± 1.96 SD) of $Pes_{V0.5}$ were -4.1 ± 5.5 cmH_2O. The $Pes_{0.5}$ - Pes_{CAL} difference inversely correlated with the $Pes_{0.5}$ - Pes_{CAL} mean value (R= -0.694, p<0.0001): the higher the Pes value, the higher the Pes underestimation due to balloon underfilling.

Panel B. Compared to calibrated Pes, bias and precision (± 1.96 SD) of $Pes_{V4.0}$ were -2.9 ± 3.3 cmH_2O. The $Pes_{4.0}$ - Pes_{CAL} difference inversely correlated with the $Pes_{4.0}$ - Pes_{CAL} mean value (R= -0.470, p<0.0001): the lower the Pes value, the higher the Pes overestimation due to the esophageal reaction to balloon inflation at 4 ml.
